# Rethinking *de novo* genes in plants: mechanisms, methodological progress, and future prospects

**DOI:** 10.3389/fpls.2025.1724832

**Published:** 2025-11-17

**Authors:** Man Luo, Haibo Wu, Dinghua Zhan, Guangcai Chen, Yunpeng Cao

**Affiliations:** 1 School of Health and Nursing, Wuchang University of Technology, Wuhan, China; 2 Guangxi Gaofeng State Owned Forest Farm, Nanning, China; 3 Guangxi Colleges and Universities Key Laboratory for Cultivation and Utilization of Subtropical Forest Plantation, College of Forestry, Guangxi University, Nanning, China; 4 Guangxi Key Laboratory of Forest Ecology and Conservation, College of Forestry, Guangxi University, Nanning, China

**Keywords:** *de novo*, mechanisms, methodological, molecular features, multi-omics

## Introduction

The emergence of novel genes represents a fundamental mechanism driving evolutionary innovation and adaptive evolution in living organisms (Xia et al., 2025). For decades, the prevailing paradigm in molecular evolution has held that new genes arise primarily through duplication and divergence of existing genes, horizontal gene transfer, or recombination events such as gene fusion and fission (Cao et al., 2024; Jiang et al., 2025; Xia et al., 2025). However, rapid advances in high-throughput sequencing and multi-species genomic data reveal that *de novo* genes (i.e. protein-coding genes arising from previously noncoding DNA) are far more common than once believed, fundamentally challenging the view that genetic novelty must originate solely from preexisting gene templates (Song et al., 2022; Cao et al., 2024; Xia et al., 2025). Initially considered evolutionary rarities or anomalies, *de novo* genes have now been identified across all domains of life, from bacteria to plants and animals (Broeils et al., 2023; Cao et al., 2024; Peng and Zhao, 2024; Xia et al., 2025). Plants, in particular, present an ideal system for studying *de novo* gene origination due to their expansive genomes, abundant non-coding regions, and high transposable element content, which collectively provide a rich substrate for the birth of novel genes (Xia et al., 2025). Recent large-scale comparative genomic studies have revealed that plant genomes harbor hundreds of lineage-specific genes lacking detectable homologs in closely related species, many of which show clear evidence of *de novo* origination from ancestral non-coding sequences (Song et al., 2022; Cao et al., 2024). The molecular signatures of plant *de novo* genes reveal intriguing patterns: they typically encode shorter proteins, lack recognizable conserved domains, and are enriched in intrinsically disordered regions (Song et al., 2022; Cao et al., 2024). While these features might appear suboptimal from a traditional protein evolution perspective, they may actually facilitate rapid functional exploration and adaptation to novel cellular contexts. Expression analyses consistently show that plant *de novo* genes exhibit highly restricted spatiotemporal patterns, often being activated only during specific developmental stages, in particular tissues, or in response to environmental stresses—suggesting fine-tuned regulatory roles in adaptive responses (Song et al., 2022; Cao et al., 2024). Population genetic evidence increasingly supports the functional importance of *de novo* genes in plant adaptation (Cao et al., 2024; Zhao et al., 2024; Li et al., 2025). Several well-characterized examples demonstrate their contributions to key biological processes: the rice *OsDR10* gene confers pathogen resistance (Xiao et al., 2009), the Arabidopsis *AtQQS* gene regulates carbon-nitrogen metabolism and enhances disease resistance (Qi et al., 2019), Rosa *SCREP* regulates eugenol biosynthesis (Li et al., 2025), and numerous other *de novo* genes have been implicated in stress tolerance, reproductive success, and developmental regulation (Zhao et al., 2024). These discoveries underscore that *de novo* genes are not merely evolutionary noise but can provide substantive adaptive benefits. Despite major advances in *de novo* gene research, key challenges persist, including the need for high-quality genome assemblies, complex phylogenetic analyses, and multi-level functional validation for accurate identification, as well as the difficulty of distinguishing true *de novo* origins from rapid sequence divergence that obscures homology (Xia et al., 2025). Moreover, determining the functional significance of putative *de novo* genes and understanding how they integrate into existing gene regulatory networks represent ongoing scientific frontiers. This opinion article examines current understanding of *de novo* gene origination mechanisms in plants, evaluates methodological advances and limitations, and discusses implications for plant evolution and potential applications in crop improvement.

## The current understanding and methodological advances in plant *de novo* gene studies

### Mechanisms of origination: genome architecture and the role of transposable elements

Plant genomes provide an exceptionally fertile ground for *de novo* gene origination due to their unique architectural features. Large-scale comparative genomic analyses across diverse plant lineages reveal that extensive noncoding regions, comprising up to 85% of some plant genomes, harbor abundant cryptic open reading frames that can potentially evolve into functional genes (Zhao et al., 2024; Xia et al., 2025). This vast noncoding landscape, combined with frequent whole-genome duplications and chromosomal rearrangements characteristic of plant evolution, creates numerous opportunities for the emergence of novel coding sequences (Zhao et al., 2024; Xia et al., 2025). Transposable elements (TEs) play a particularly crucial role as catalysts for *de novo* gene birth in plants (Jin et al., 2021b; Zhao et al., 2024; Xia et al., 2025). Recent evidence demonstrates that TEs, which constitute 45-85% of many plant genomes, actively facilitate gene origination through multiple mechanisms (Jiang et al., 2022; Pulido and Casacuberta, 2023; Cao et al., 2025). First, TE insertions can directly provide promoters, enhancers, and transcription factor binding sites that activate transcription of nearby noncoding sequences. Second, TEs mediate chromosomal rearrangements that bring together previously separated noncoding fragments, creating novel transcriptional units. Third, TE-induced epigenetic modifications can establish new chromatin states conducive to gene expression (Li et al., 2025; Xia et al., 2025). Analysis of rice, maize, and Arabidopsis genomes reveals that approximately 30-40% of recently originated *de novo* genes show clear associations with TE activity, either through direct sequence contribution or regulatory element donation (Xia et al., 2025). This TE-mediated mechanism appears particularly active during periods of environmental stress or genomic instability, potentially accelerating adaptive evolution through rapid gene innovation.

### Molecular features: small, unstable proteins for rapid testing

Plant *de novo* genes exhibit distinctive molecular signatures that facilitate rapid functional exploration. These genes typically encode remarkably short proteins, often less than 100 amino acids, with high intrinsic disorder content and lacking recognizable conserved domains (Song et al., 2022; Cao et al., 2024; Xia et al., 2025). This structural “permissiveness” appears advantageous rather than detrimental—the abundance of disordered regions allows *de novo* proteins to escape strict folding constraints that govern canonical proteins, enabling them to act as flexible molecular probes capable of transient interactions and regulatory fine-tuning (Patiou et al., 2025; Xia et al., 2025). Studies in rice, Arabidopsis, and other plants consistently show that *de novo* proteins have lower intrinsic structural disorder (ISD) values, reduced GC content, and fewer secondary structure elements compared to conserved genes (Song et al., 2022; Cao et al., 2024; Peng and Zhao, 2024; Patiou et al., 2025). These properties enable rapid evolutionary testing of novel biochemical functions while minimizing the risk of misfolding and aggregation, essentially providing plants with a low-cost experimental platform for molecular innovation under selective pressures (Xia et al., 2025).

### Expression and selective fate: evidence from population genomics and functional screens

Population genomic data suggest that plant *de novo* genes exhibit sharply restricted spatiotemporal expression, being chiefly induced during reproductive development or in response to environmental challenges like drought, pathogen exposure, and nutrient deficiency (Cao et al., 2024; Jiang et al., 2025; Xia et al., 2025). Large-scale transcriptomic surveys demonstrate that while most *de novo* genes display low expression levels compared to conserved genes, they show significant tissue specificity, with enrichment in reproductive tissues, suggesting roles as molecular fine-tuners of adaptive responses (Jin et al., 2021a; Song et al., 2022; Cao et al., 2024). Selection-signature analyses (e.g., dN/dS ratios and population frequency distributions) show that *de novo* genes follow diverse evolutionary trajectories, with many genes (especially those involved in stress response and reproduction) being subject to positive or balancing selection (Kaessmann, 2010; Song et al., 2022; Cao et al., 2024; Xia et al., 2025). In addition, population studies also find that about 25%-30% of young genes become essential, such that their silencing is lethal (Li et al., 2025). However, many *de novo* genes are rapidly lost through genetic drift or negative selection, reflecting an ongoing evolutionary “trial-and-error” process (Van Oss and Carvunis, 2019; Xia et al., 2025). Functional validation through knockout experiments and CRISPR screens confirms that some *de novo* genes provide genuine adaptive advantages, such as the rice *OsDR10* conferring pathogen resistance and Arabidopsis *AtQQS* regulating metabolic networks (Xiao et al., 2009; Tanvir et al., 2022). Nevertheless, distinguishing truly functional *de novo* genes from transcriptional noise remains challenging, requiring convergent evidence from genomics, transcriptomics, proteomics, and experimental validation.

### Methodological innovations: comparative genomics, multi-omics, and integrative frameworks

Recent methodological advances have revolutionized plant *de novo* gene identification and characterization. Progressive whole-genome alignment tools like Cactus now enable high-confidence synteny-based identification across divergent species, surpassing traditional BLAST-based approaches (Li et al., 2025; Xia et al., 2025). Multi-omics integration combining RNA-seq, Ribo-seq, proteomics, and metabolomics provides convergent evidence for gene functionality, addressing the challenge of distinguishing genuine *de novo* genes from transcriptional noise (Jin et al., 2021a; Song et al., 2022; Cao et al., 2024). Advanced computational frameworks incorporating deep learning (AlphaFold2) predict protein structures, revealing that some *de novo* proteins can achieve well-folded conformations despite lacking conserved domains (Li et al., 2025). Weighted gene co-expression network analysis (WGCNA) demonstrates how *de novo* genes integrate into existing regulatory networks (Jin et al., 2021a; Cao et al., 2024). Population genomics approaches using dN/dS ratios and selection signatures reveal adaptive evolution patterns (Song et al., 2022; Cao et al., 2024). These integrative pipelines, combining phylostratigraphy, expression profiling, and functional validation through CRISPR/Cas9, establish robust standards for *de novo* gene annotation and functional characterization in plants (Song et al., 2022; Li et al., 2025; Xia et al., 2025).

### Challenges and hypotheses: strengths and weaknesses

Despite these advances, several unresolved problems demand attention. First, annotation errors and incomplete genome assemblies (especially widespread in polyploid and repetitive plant genomes) affect the accuracy of gene age assignment and detection sensitivity (Xia et al., 2025). Second, phylostratigraphic approaches can overestimate *de novo* birth by failing to detect highly divergent homologs, while excessive stringency risks false negatives (Van Oss and Carvunis, 2019; Peng and Zhao, 2024). Third, not all detected ORFs possess biological function; some may reflect pervasive translation “noise,” and distinguishing functional *de novo* genes from translation byproducts remains technically challenging (Peng and Zhao, 2024; Xia et al., 2025). Misinterpretation is also a concern—identifying a recently fixed gene does not alone imply strong adaptive value, and function must still be established by knockout, phenotyping, or pathway analysis. The standards for *de novo* gene proof thus continue to shift toward convergence of evidence from genomics, transcriptomics, proteomics, and experimental approaches (Peng and Zhao, 2024).

The “proto-gene continuum” hypothesis, positing a spectrum from spurious ORFs to fully-fledged new genes, finds support in plant datasets: only a fraction of new sequences escape rapid loss, often after acquiring beneficial regulatory context through TE activity or environmental induction (Van Oss and Carvunis, 2019; Xia et al., 2025). Plant studies particularly demonstrate how the noncoding genome acts as a reservoir for rapid trait innovation, especially under strong selection or in adaptive radiations (Zhao et al., 2024; Xia et al., 2025). Importantly, recent research reveals the potential role of epigenetic state and regulatory plasticity in facilitating or constraining *de novo* gene emergence—topics that are rapidly gaining ground in the literature (Zhao et al., 2024; Li et al., 2025). Nevertheless, the field would benefit from more careful functional dissection, especially for genes found mostly in single accessions or populations. Scientific caution and explicit reporting of uncertainty are crucial to avoid over-attributing functions to recently emerged ORFs.

## Discussion

Recent research has clarified the significant contribution of *de novo* genes to plant evolution and adaptation, although substantial challenges and open questions remain (Cao et al., 2024; Li et al., 2025). Plant genomes, rich in noncoding DNA and transposable elements, are particularly conducive to the emergence of new genes from previously noncoding regions (Tao et al., 2025). Transposable elements (TEs) create new genes through two primary mechanisms. First, they can generate genes from non-coding DNA by providing regulatory elements, like promoters, that activate adjacent sequences, or through the “exonization” of their own sequences. Second, they can modify existing genes. This occurs when a TE’s own gene is “molecularly domesticated” for a novel host function, as with the *RAG1* gene (Agrawal et al., 1998), or when TE insertion fuses host genes to create a new chimeric gene. Evidence shows that plant *de novo* genes often have highly specific expression and are rapidly induced by stress or distinct developmental processes (Zhao et al., 2024; Tóth et al., 2025; Xia et al., 2025), supporting the idea that they constitute a flexible, fast-evolving toolkit that helps plants handle novel environmental challenges. However, interpreting the functional impact of these genes requires caution. While experimental and population genetic studies reveal that some *de novo* genes can provide adaptive advantages, the majority of candidate *de novo* genes remain uncharacterized or may even represent evolutionary transient entities. Distinguishing between genuinely functional *de novo* genes and those which are byproducts of pervasive translation remains an ongoing difficulty. Functional validation through knockout or overexpression studies is still available for only a minority of plant *de novo* genes (Xia et al., 2025).

Methodologically, the field continues to face significant obstacles, particularly in the annotation and validation of *de novo* gene candidates. High rates of sequence divergence, polyploidy, and limited genome annotation quality in many plant species can result in both false positives and false negatives when identifying and age-dating *de novo* genes (Xie et al., 2024; Xia et al., 2025). Recent advances such as deep co-linearity analysis, integrated multi-omics, and large-scale phylogenetic sampling all help to increase confidence, but robust community standards are still needed for declaring bona fide *de novo* genes (Xia et al., 2025). Despite these hurdles, ongoing research is moving towards a more nuanced view of genome innovation, where the *de novo* gene birth is not a rare accident, but a recurrent source of biological novelty. As functional genomics tools advance, systematic exploration of *de novo* gene roles in phenotypic traits, stress responses, and crop improvement will become increasingly feasible. Ultimately, integrating evolutionary, genomic, and ecological perspectives will be essential to fully understand the frequency, impact, and practical utility of *de novo* genes in plants. Future work should prioritize (1) standardized, multi-tier evidence frameworks reporting the status and confidence of candidate *de novo* genes, (2) integration of ecological, population, and molecular genetics, and (3) experimentally assessing the impact of *de novo* gene emergence on plant fitness and adaptation. In this way, plant *de novo* gene science can provide not only theoretical advances but also practical tools for sustainable agriculture and biological understanding.
